# Designing complex health system interventions: an integrated theory-informed approach

**DOI:** 10.3389/frhs.2026.1820296

**Published:** 2026-07-09

**Authors:** Shobhana Nagraj, Francesca Maraschin

**Affiliations:** 1 Department of Public Health and Primary Care, University of Cambridge, Cambridge, United Kingdom; 2 East London NHS Foundation Trust, London, United Kingdom

**Keywords:** behaviour change, health systems, implementation science, systems thinking, theory-informed design

## Abstract

Health systems interventions are increasingly designed to address complex challenges in quality, safety, access, and equity. Yet many fail to achieve sustained impact, often due to misalignment between intervention mechanisms, organisational context, and broader system dynamics. Although theory is widely promoted to strengthen intervention design, frameworks from behavioural science, implementation science, and systems thinking are frequently applied in isolation, limiting their collective explanatory and practical value. This focused narrative review adopts a design-first perspective—defined as the prospective use of theory to inform intervention development rather than its retrospective use to explain outcomes, to synthesise and compare key theoretical approaches used to inform the development of complex health system interventions. Drawing on a narrative synthesis of 46 review articles (33 identified through database searching and 13 through citation chasing of foundational sources) published between 2010 and 2026, we examine how behavioural science, implementation science theories and frameworks, and systems thinking approaches contribute complementary insights across the micro- (individual/interpersonal), meso- (team/organisational) and macro- (system/policy) levels of complexity. We argue that effective intervention development requires a layered design architecture that explicitly aligns micro-level behavioural mechanisms, meso-level organisational and implementation processes, and macro-level system dynamics. We also argue that the boundaries between these traditions are increasingly fluid, and that hybrid frameworks and the integration of design thinking with implementation and systems science, represent a particularly fertile area for advancing intervention design. By integrating these traditions, we propose a pluralistic and structured approach to intervention design that strengthens causal clarity while accommodating adaptation and contextual variability. Advancing such integrative practice is essential for improving the effectiveness, scalability, and sustainability of health system interventions.

## 1 Introduction

Health systems are complex adaptive systems, that encompass a combination of people, institutions, resources, and policies designed to promote, restore, and maintain the health of a population (1). They comprise heterogeneous actors, including patients, clinicians, managers, commissioners, regulators, professional bodies and policy-makers, with differing incentives, power, professional norms, time horizons, and authority (2, 3). They are characterised by non-linearity, feedback loops, and emergent behaviour. Health systems worldwide invest heavily in interventions aimed at improving quality, safety, access, and equity of care. These interventions typically comprise multiple interacting components, operate across organisational and professional boundaries, and are embedded within dynamic social and political contexts (4, 5). Interventions introduced into such systems are rarely implemented as designed; instead, they are adapted, resisted, or repurposed by individuals and organisations in response to local constraints and priorities (6, 7).

In this review, we define health system interventions as multi-component strategies designed to influence healthcare delivery, organisation, financing, or policy, and which typically span more than one level of the health system. We use the term “level” to refer to: the micro-level (individual patient, clinician, or interpersonal interaction); the meso-level (clinical team, service, or organisation); and the macro-level (sector, policy, or wider health system). The review focuses on interventions delivered within or by health and care services, rather than on broader population-health interventions delivered outside the healthcare sector.

The UK Medical Research Council (MRC) describes complex interventions as those involving multiple interacting components, variability in outcomes, and sensitivity to context (4). Complexity, however, extends beyond the number of components to include dynamic interactions among actors, organisational processes, and system structures over time (3). Many health system interventions target multiple behaviours across professional groups, operate across organisational layers, and are influenced by policy-level drivers. Wider socio-political, cultural, and infrastructural conditions further compound these dynamics (7). The 2021 update of the MRC framework (4), together with the MRC ADAPT guidance (5), explicitly emphasises the iterative use of programme theory, stakeholder engagement, and contextual adaptation across the design and evaluation lifecycle.

Theory is widely advocated as a mechanism to strengthen intervention design by clarifying causal assumptions, identifying mechanisms of change, and supporting implementation and evaluation (8–12). Despite growing emphasis on theory-informed development, many interventions fail to achieve intended outcomes, demonstrate limited transferability, or show variable sustainability across settings (13, 14). Furthermore, in practice, theory is often applied either superficially or in isolation (13, 15). Single frameworks are frequently expected to fulfil multiple functions—from problem diagnosis and intervention specification to implementation planning and evaluation, leading to either theoretical overload or conceptual dilution (13). This persistent gap between theoretical promise and real-world impact underscores the challenges of designing interventions that are not only effective, but also feasible, scalable, and adaptive within complex health system environments (5, 16).

No single theoretical approach adequately captures the full spectrum of complexity inherent in a health system. Intervention design therefore may benefit from theoretical pluralism: the deliberate integration of complementary approaches drawn from behavioural science, implementation science, and systems thinking. Each offers distinct analytical strengths. Despite their complementary strengths, these approaches are rarely integrated systematically during the intervention design phase.

A key source of confusion in the literature is the distinction between two related, but conceptually distinct uses of theory: a) theory to specify intervention content and mechanisms of action (e.g., behaviour change theories), and b) theory to guide the overall process of intervention development and implementation within complex systems (e.g., implementation/complex intervention frameworks). These two uses of theory operate at different conceptual levels and answer different questions: the first asks “what should the intervention do?”, the second asks “how should the intervention be developed, embedded and adapted?”. Both are essential, but they are often used in isolation. This review treats both functions of theory in parallel and considers how they can be integrated within a single design architecture.

This focused narrative review adopts a design-first lens, which we define as the prospective use of theory to inform intervention development**—**including problem framing, mechanism specification, contextual fit, and anticipation of system effects. We view this as distinct from the retrospective use of theory to explain why an intervention succeeded or failed after implementation. We synthesise a selection of the contemporary literature spanning behavioural science, implementation science, and systems thinking, to examine how these traditions contribute to the design of complex health system interventions, identify gaps in their integration, and propose a structured, multi-level approach to theory-informed design.

## 2 Methods

We conducted a focused narrative literature review (consistent with the journal's mini-review article type), to synthesise theoretical and methodological approaches used to inform the design of complex health system interventions. This approach was selected (rather than a systematic or scoping review), to enable conceptual integration across diverse theoretical traditions, rather than to provide a comprehensive systematic mapping of all available frameworks.

A structured, but non-exhaustive literature search was conducted in PubMed and Google using combinations of terms related to “health systems,” “healthcare,” or “health services” with “interventions,” and “models,” “frameworks,” or “theories.” Searches were limited to English-language review articles published between 2010 and 2026, and included systematic, scoping, narrative, and realist reviews. Consistent with the integrative and conceptual aims of this review, the search strategy prioritised widely applied theoretical approaches shaping contemporary intervention design (2010–2026) across behavioural science, implementation science and systems thinking. We operationalised “intervention design” as the use of theory, models, or frameworks (TMF) to inform problem framing, intervention specification, implementation strategy selection, or any combination of these, prior to or during the development of a complex health system intervention.

The search yielded 2,388 records. Titles and abstracts were screened independently by two authors (SN, FM) against the inclusion criteria. Full-text articles were assessed by both authors and disagreements resolved through discussion. After excluding abstracts, opinion pieces, and reviews not explicitly addressing complex health system interventions or theoretical approaches to intervention design, 33 papers were included. Citation and targeted hand-searching identified an additional 13 papers, resulting in a total of 46 included studies (see Supplementary Appendix 1 for PRISMA-style flow diagram and Data extraction table). These papers were then contextualised within the wider literature of foundational and highly cited frameworks.

## 3 Results

We summarise three main approaches which currently dominate thinking on health system intervention design for analytic clarity. In practice, the boundaries between these approaches are fluid. The challenges, limitations and integrative implications of these approaches are synthesised further in the Discussion, as they represent a particularly important emerging area of work.

Supplementary Table 1 summarises the principal TMFs discussed in the review and situated within the foundational literature. These are organised by theoretical tradition, level of action, and the way each operationalises complexity.

### 3.1 Behaviour change theories, models and frameworks: specifying mechanisms

Behaviour change theories provide structured methods for identifying determinants of practice and specifying intervention components. Models and frameworks such as the COM-B model and the Behaviour Change Wheel (BCW) (17) enable systematic analysis of capability, opportunity, and motivation as key drivers of human behaviour. Complementary tools such as the Theoretical Domains Framework (TDF) (18) integrate classic motivational theories including the Theory of Planned Behaviour (19) (emphasising attitudes, subjective norms, and perceived behavioural control), Social Cognitive Theory (20) (highlighting self-efficacy and observational learning), the Health Belief Model (21) (focusing on perceived susceptibility, severity, benefits and barriers), and the Transtheoretical Model (22), which describes behaviour change as a stage-based process from pre-contemplation through to maintenance. Used together with the Behaviour Change Technique (BCT) Taxonomy (23), these theories, models and frameworks support transparent specification of mechanisms and intervention content, addressing longstanding concerns around the lack of transparency, evidence base, and theoretical underpinnings guiding health system intervention development and subsequent evaluation.

Behaviour change theories and frameworks have been widely applied across clinical, community and digital settings. Rather than presenting individual applications as discrete examples, we organise them here into three modes of application that emerged from our synthesis. First, retrospective synthesis: frameworks such as COM-B and the BCT Taxonomy were used to code barriers, facilitators, or intervention content across populations (24–26). Second, prospective intervention specification: the BCW is used to structure clinical interactions and digital prompts (27). Third, effectiveness and optimisation evaluation: intervention design features and BCTs were linked to outcomes over time (28, 29). Together, these modes form a continuum from descriptive synthesis through to system-level implementation and evaluation, rather than a random collection of applications.

Systematic and scoping reviews demonstrate increasing use of the COM-B model and the BCW to inform intervention development, particularly for physical activity (24–28) and professional practice change (29). For example, rapid and systematic reviews applying COM-B and BCT mapping to musculoskeletal physical activity barriers (24), and to breast cancer survivorship interventions (25), respectively, highlight how systematic theoretical analysis may improve alignment between needs assessment and proposed intervention functions and techniques. Hand hygiene interventions in community settings increasingly specify BCTs linked to theoretical determinants (30).

Applications of the TDF further demonstrate the role of theory in structuring healthcare professional behaviour change (31, 32), while reviews of continuing professional development research highlight inconsistent and often superficial theory use (33). This uneven application reinforces concerns raised in broader methodological syntheses that behavioural theory is frequently invoked, but insufficiently integrated into broader system design (26, 34).

Importantly, behaviour change approaches have also been embedded within participatory and person-centred design methods. The Person-Based Approach (35) and experience-based and human-centred co-design initiatives (36–38) illustrate how behavioural specification can be combined with user engagement to enhance intervention acceptability and feasibility. However, these approaches often remain focused at the micro-level, with limited explicit integration of organisational or system dynamics, and may not always be reported. Concerns regarding reporting quality and intervention specification persist, as highlighted by the Workgroup for Intervention Development and Evaluation Research (WIDER) recommendations (39).

Behavioural diagnoses to inform intervention design can be conducted at the macro-, meso- and micro-levels of the health system. At the macro-level, behavioural theory can be applied to the decisions of policymakers, commissioners and senior leaders, whose choices shape resource allocation, regulation, and the conditions for clinical and organisational change. At the meso-level, behavioural theory applies to clinical teams and the wider workforce. At the micro-level, it applies to individual patients and clinicians. Our mini review highlighted that behaviour change TMFs were predominantly used at the micro-level, with limited evidence of their use to inform intervention design at the macro-level. This is a notable gap.

Decision-makers operate under diverse incentives, accountability structures, time horizons, political constraints and competing priorities, all of which make their behaviour both highly consequential for system performance and difficult to influence through interventions designed only with frontline actors or end-users in mind. Extending behaviour change frameworks (including COM-B, TDF, and the Transtheoretical Model) to systematically inform the design of policy and managerial interventions, in dialogue with implementation and systems perspectives, represents a high-priority area for future work.

The strength of behaviour change approaches within intervention design lies in their mechanistic clarity and practical usability. By explicitly linking behavioural determinants to intervention components, these TMFs facilitate the development of interventions that are theoretically coherent and replicable. Such approaches are particularly valuable during early design stages, such as problem definition and intervention specification.

Behaviour change theories are often grounded in relatively linear models of causation and tend to privilege individual-level explanations of behaviour. In many global contexts, with less individually focused lifestyles, decision-making is collective and occurs in the context of wider social groups and communities (40). When applied to complex health systems interventions, this focus may underplay organisational dynamics, professional hierarchies, and broader system constraints, such as gender-related factors impacting health system access and the socio-political environment in which the interventions function.

### 3.2 Implementation science frameworks: addressing context and organisational dynamics

To date, implementation frameworks have frequently been applied retrospectively, serving primarily to explain variation in outcomes rather than proactively shaping intervention design (41, 42). Although widely used implementation frameworks such as the Consolidated Framework for Implementation Research (CFIR) (43), the Exploration, Preparation, Implementation, Sustainment (EPIS) framework (44), the Reach, Effectiveness, Adoption, Implementation, Maintenance (RE-AIM) framework (45), and the integrated Promoting Action on Research Implementation in Health Services (i-PARIHS) framework (46) offer comprehensive classifications of contextual determinants, they often function as descriptive taxonomies rather than prescriptive design tools. Reviews have highlighted that these frameworks provide limited operational guidance for translating identified barriers and enablers into specific, testable intervention components (34, 44). As a result, their utility during early-stage intervention development, where mechanisms of action and intervention component selection must be specified, can be constrained.

Methodological guidance on complex intervention development increasingly calls for more structured and prospective theory integration, including explicit articulation of programme theory and iterative adaptation across system levels (47, 48), enabling “learning” within the health system. Emerging de-implementation research further underscores the need for theoretically informed strategies that address not only uptake but also the removal, replacement, or modification of low-value practices within dynamic service environments (49). Collectively, this body of work suggests that while determinant frameworks are valuable for contextual diagnosis, additional methodological scaffolding is required to bridge the gap between contextual analysis and intervention specification.

Implementation science extends this analytical lens to organisational and contextual determinants of adoption and sustainability. Normalization Process Theory (NPT), a mid-range implementation theory, highlights the importance of professional relationships, hierarchies, workflow integration and collective action in embedding practices into routine care (50). NPT illuminates why interventions that are mechanistically coherent may nevertheless fail due to misalignment with organisational capacity, leadership engagement or contextual readiness. In their systematic review, May et al. (42) examined how NPT had been used in feasibility studies and process evaluations of complex healthcare interventions. They showed that prospective application of the four NPT constructs—coherence (how the intervention is understood by those involved), cognitive participation (engagement and ownership), collective action (practical integration into workflows) and reflexive monitoring (ongoing appraisal and adaptation), might enable researchers to identify and reduce cognitive burden, clarify role expectations across clinicians and facilitators, and restructure intervention components to align with routine clinical pathways. Combined use of CFIR and the Theoretical Domains Framework (TDF) further bridges behavioural and contextual determinants within a unified analytic strategy (51).

In parallel, CFIR has been used prospectively to systematically assess determinants of implementation across inner setting (structural and cultural attributes of the organisation), outer setting (external policy and resource environment) and intervention characteristics domains, informing adaptations such as flexible delivery formats and embedded implementation supports (43). Barnden et al., in a systematic review of prospective implementation framework use in hospital settings, demonstrated that this approach may enhance design quality, stakeholder engagement and uptake, and is consistent with wider implementation science practice (52). The combined use of CFIR with complementary implementation frameworks has been widely recommended as a strategy to capture multi-level determinants of implementation, reflecting the increasing methodological pluralism of the field (51). Together, NPT and CFIR might enable a prospective, theory-informed optimisation process that actively shapes feasibility, acceptability, and potential for routine integration within clinical services.

Recent syntheses extend this perspective to questions of scalability and system alignment. Klaic et al. conducted an overview of reviews and developed a conceptual framework for “implementability” (the combined construct of acceptability, feasibility, fidelity, and sustainability), and provided practical guidance for assessing and building these properties into complex intervention design prior to a trial (53). Gilhooly et al. reviewed the development, implementation, and evaluation of care bundles in acute hospital care and found that successful implementation was contingent on organisational alignment, leadership support and workflow integration rather than on technical specification alone (54). These reviews point towards the importance of wider factors influencing intervention success and sustainability. In global primary and community care contexts for example, both intervention content and wider support architecture shape healthcare worker performance. Kok et al. identified supervision, incentives, governance, and training as key determinants of CHW effectiveness (55), while Vasan et al. demonstrated that supervision, mentoring, tools, and quality improvement interventions frequently overlap in practice and are difficult to disentangle as discrete categories (56). Cairns et al. scoping review of healthcare worker wellbeing interventions during the COVID-19 pandemic, further reinforce the importance of organisational and policy-level responsiveness in sustaining intervention impact under conditions of acute system disruption (57).

However, even when applied prospectively, implementation frameworks often assume relatively bounded and stable organisational contexts. They typically conceptualise determinants as discrete variables rather than as interacting elements within adaptive systems. This limitation becomes particularly evident when considering complex health systems characterised by feedback loops, non-linearity, political drivers and evolving institutional arrangements. While implementation science has advanced understanding of contextual determinants and organisational embedding, it may not always fully capture emergent system dynamics.

These limitations point toward the added value of systems thinking and complexity science. Whereas behaviour change models emphasise individual mechanisms and implementation theories foreground contextual determinants, systems approaches conceptualise interventions as events introduced into dynamic, adaptive systems. They draw attention to feedback loops, path dependency, power relations, cross-level interactions, and unintended consequences—features that may shape both implementation processes and long-term sustainability.

### 3.3 Systems thinking and complexity-informed design: emergent properties of systems

Systems thinking introduces tools and theories that address complexity more explicitly. Approaches such as System Dynamics (58), agent-based modelling (59), and realist methods (60) conceptualise interventions within evolving systems characterised by feedback, adaptation, and emergence. Systems thinking and complexity science provide a distinct lens, emphasising non-linearity, adaptation, feedback loops and emergent behaviour (61). Interventions are conceptualised as events interacting dynamically with system structures and processes rather than isolated inputs (62). Systems thinking approaches support problem framing, boundary-setting, and identification of leverage points for health system interventions.

Practical models that operationalise systems thinking during design and evaluation include the Systems Engineering Initiative for Patient Safety (SEIPS) framework (63), which models healthcare work as a sociotechnical system of people, tasks, tools, environment and organisation, and the Engineering Better Care (EBC) framework (64) developed by the Royal Academy of Engineering, which adapts systems engineering principles for health and care system design. Both rely heavily on systems mapping—the use of techniques such as flowcharts and causal loop diagrams to convert complex information about a health system into a shared visual representation, typically in collaboration with stakeholders (63, 64). Despite their relevance, systems thinking methods are often applied conceptually rather than operationally, reflecting methodological gaps and capacity constraints. These approaches support anticipation of unintended consequences, policy resistance, and dynamic adaptation over time. However, systems approaches sometimes lack the behavioural precision required to specify concrete intervention components or the organisational granularity necessary for planning implementation and may benefit from being used in synergy with behaviour change approaches and implementation science (65, 66).

Systems-oriented approaches increasingly recognise that interventions occur within adaptive systems rather than as discrete packages. Further guidance on complex interventions emphasises iteration, programme theory and context sensitivity (5, 47, 48). Realist-informed organisational reviews (67) and health systems theory applications (68) demonstrate how outcomes emerge from the interactions between context–mechanism–outcome configurations rather than by linear causality.

Participatory and co-design methodologies, including community-based participatory research (69), stakeholder-driven inequity frameworks (70), and deliberative dialogue models (71), reflect a systems recognition that intervention legitimacy and sustainability depend on distributed agency across system actors. Recent work also demonstrates the potential of embedding participatory systems modelling and group model building, as practical tools for building stakeholder capacity to “think in systems” and enhance the implementation of public health interventions (72). Research on digital health interventions further highlights systemic inequities and adaptive challenges. Reviews addressing the digital divide show that interventions designed without attention to structural access, literacy and infrastructural capacity, risk exacerbating inequities (73–75). These findings reinforce the need for embedding systems thinking principles of feedback, path dependency and unintended consequences into intervention design. Design thinking methodologies (76) and adaptive frameworks such as the Approach to Human-Centered, Evidence-Driven Adaptive Design (AHEAD) (38) similarly foreground iterative prototyping and responsiveness to emergent system behaviour. Building on this, Huang et al. propose a framework that explicitly integrates systems science and design thinking to advance implementation science, arguing that this integration moves the field from a problem-oriented to a solution-oriented paradigm (77).

## 4 Discussion

This focused narrative review synthesises three broad theoretical traditions that inform the design of complex health system interventions. Although we present these three traditions separately for analytic clarity, several useful TMFs for design sit explicitly in this zone of overlap. Examples include the combined use of CFIR and TDF, which bridges contextual and behavioural determinants (51), the extension of COM-B into socio-ecological systems (SeCOM-B) (40), integration of systems thinking with implementation science (66, 77); and with design thinking (77). This convergence suggests that the most productive direction for the field is not the parallel application of three separate traditions but the deliberate cultivation of the conceptual space they share, and the development of design practices, hybrid frameworks and pluralistic logic models that operate across that space (see Box 1).

Box 1 **A six-step integrated design sequence.**

1. **Problem framing (systems lens):** Define system boundaries, key actors, dynamic interactions, feedback loops and power relations. Tools: systems mapping, causal loop diagrams, stakeholder analysis (drawing on SEIPS (63), EBC (64).
2. **Behavioural diagnosis (behavioural lens):** Identify target behaviours across actors (patients, clinicians, managers, decision-makers) and specify underlying determinants. Tools: COM-B & BCW (17), TDF (18), Transtheoretical Model (22), BCT Taxonomy (23).
3. **Contextual mapping (implementation lens):** Assess organisational readiness, leadership, workflows, and contextual constraints influencing adoption. Tools: CFIR (43), NPT (6), EPIS (44), i-PARIHS (46).
4. **Intervention specification (integration):** Align BCTs with contextual realities, ensuring mechanisms are feasible within organisational settings and consistent with system-level dynamics. Tools: programme theory, logic model, theory of change; combined CFIR–TDF; SeCOM-B (40).
5. **System alignment (systems lens):** Anticipate system-level interactions, unintended consequences, and adaptation over time. Tools: realist context–mechanism–outcome configurations, system dynamics, agent-based modelling.
6. **Iterative adaptation (cross lens):** Use ongoing learning, feedback, and stakeholder input to refine the intervention while maintaining core mechanisms. Tools: AHEAD (38), Person-Based Approach (35), MRC ADAPT guidance (5).

**Worked example:** A primary-care pathway intervention might combine systems mapping of referral flows, behavioural diagnosis of clinician use, organisational readiness assessment using CFIR, specification of BCT-informed implementation supports, and iterative adaptation informed by system feedback.

The integration of behavioural, implementation and systems perspectives can be operationalised through programme theory development, including logic models and theories of change, which explicitly link mechanisms, contextual conditions, and system dynamics across levels. The MRC framework (4) and the MRC ADAPT guidance (5) provide complementary scaffolding for this work, with ADAPT emphasising context-sensitive adaptation as an intrinsic part of design.

### 4.1 An integrated, multi-level design architecture

We propose conceptualising intervention design as a layered architecture spanning the micro-, meso- and macro-levels of the health system, in which different theoretical lenses are applied deliberately at different stages and levels rather than sequentially or in isolation. At the micro-level, behaviour change theories enable precise specification of mechanisms targeting individual and interpersonal behaviours. At the meso-level, implementation science frameworks guide organisational alignment, leadership engagement and contextual readiness to ensure adoption and feasibility. At the macro-level, systems thinking approaches address dynamic feedback, adaptation, and policy-level influences that shape sustainability and scale.

Effective design requires explicit alignment across and between these levels. Behavioural mechanisms must be operationally feasible within organisational structures; implementation strategies must account for system feedback; and system-level adaptations must preserve core mechanisms of change. Misalignment can lead to interventions that are theoretically coherent but operationally unworkable, or contextually feasible but mechanistically weak.

**Figure 1:**
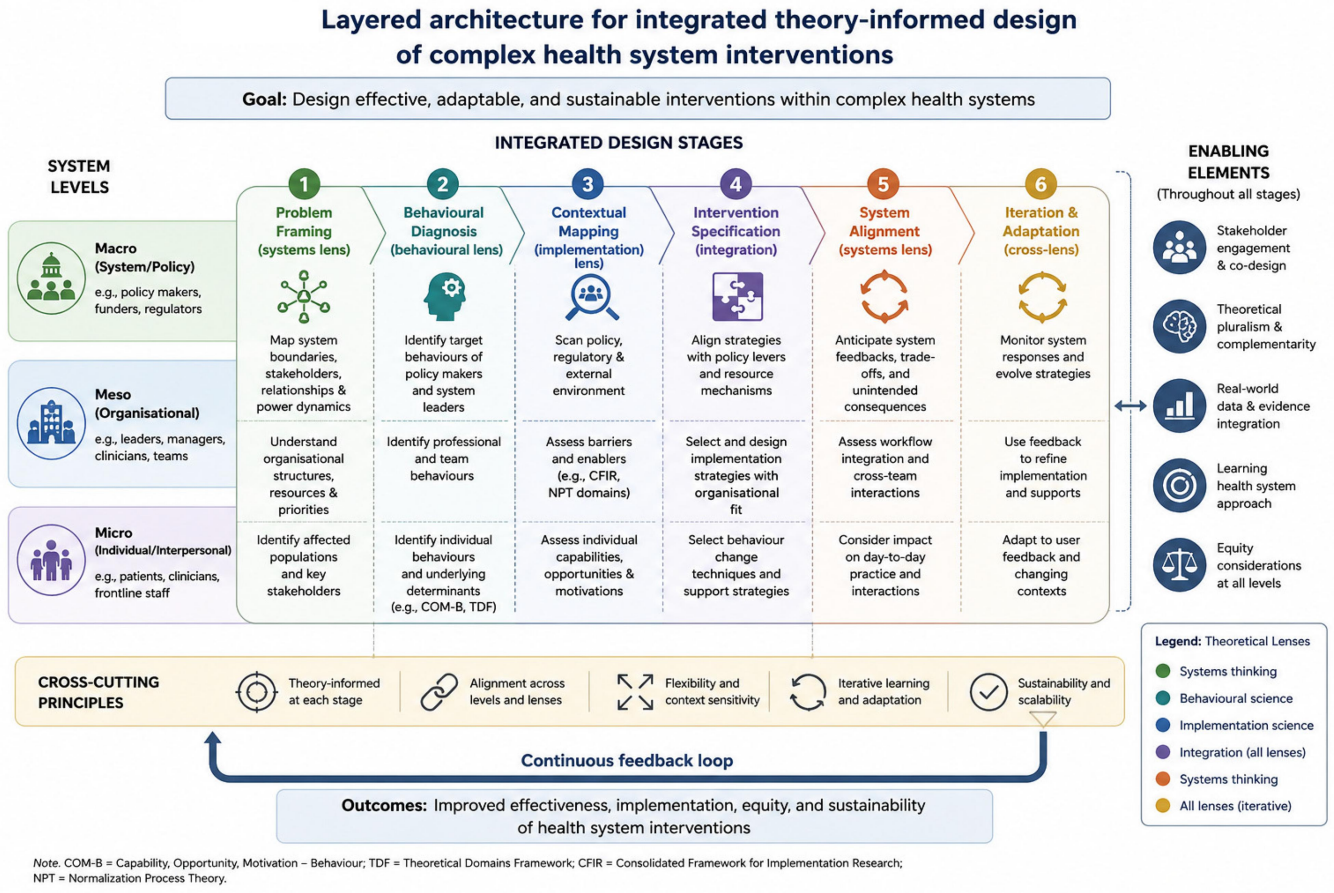
Layered architecture for integrated theory-informed intervention design.

This pluralistic, layered approach strengthens causal clarity while accommodating adaptation and contextual variability, providing a robust foundation for health system transformation. Figure 1 presents the layered architecture visually.

### 4.2 Limitations

This focused narrative review has several limitations. Coverage is non-exhaustive, the search was limited to PubMed and Google and the synthesis prioritises conceptual integration over comprehensive mapping. The proposed integrated architecture is conceptual rather than empirically tested, and empirical case studies of fully integrated, design-first applications of all three traditions remain rare, a point we return to in Future Directions.

### 4.3 Future directions

Advancing complex health system intervention design requires increased methodological attention to integration, transparency, and adaptability. Hybrid approaches that leverage multiple theoretical perspectives across distinct design stages are particularly promising. Priority areas include: (i) the systematic application of behavioural frameworks to meso-level decision-makers and policy actors, not only frontline clinicians; (ii) the development of empirical case studies that demonstrate the integrated, multi-level architecture in practice; (iii) further development of hybrid frameworks; and (iv) capacity-building initiatives to equip interdisciplinary teams with the skills required to apply theory across multiple levels and perspectives. By adopting a design-first, cross-theoretical approach, future research can better support the development of interventions that are effective, feasible and sustainable within complex adaptive health systems.

## 5 Conclusion

Designing interventions in complex health systems necessitates approaches that integrate behavioural, implementation and systems perspectives. While each offers valuable insights, no single framework is sufficient. A design-first, theoretically plural approach, oriented around the conceptual space these traditions share, rather than around their separation, may improve feasibility, adaptability, and sustainability, bridging the gap between theoretical knowledge and real-world implementation, and supporting transformative health system change.
