# Supplementary material for: Designing complex health system interventions: an integrated theory-informed approach
Source: Front Health Serv. 2026 Jul 9;6:1820296. doi: 10.3389/frhs.2026.1820296 (PMC13391582; doi:10.3389/frhs.2026.1820296)
Supplement: Supplementary file 1 [file Datasheet1.pdf]

Appendix 1: Supplementary Material

Search strategy

("health system"[Title/Abstract] OR "health service"[Title/Abstract] OR "healthcare"[Title/Abstract]) AND "intervention"[Title/Abstract] AND ("model"[Title/Abstract] OR "theory"[Title/Abstract] OR "framework"[Title/Abstract])

Filters:

- Reviews, systematic reviews, scoping review
- English
- 2010/1/1 – 2026/1/30

Final search strategy: 2,388
Included papers for title abstract: 110
Included after full text screening: 33
Forward/backward citation: 13
Final included: 46

Prisma style flow chart of Database and citation searches

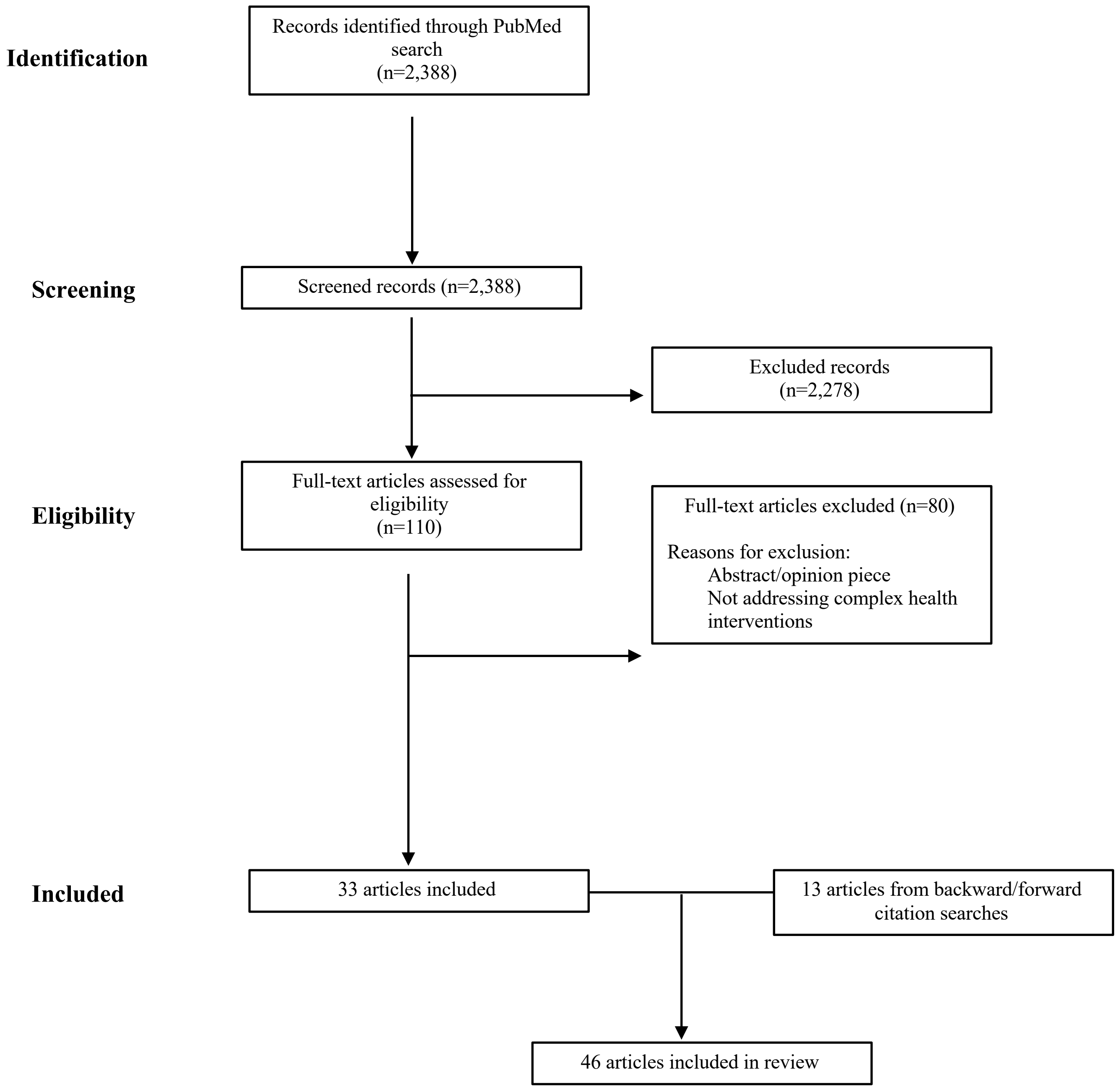

Table 1.1: Summary of studies included in mini review: Theories, Models & Frameworks in Health Systems (n=46)

Author(s) & Year	Title	Type of Review	Primary Theoretical Approach	Key Findings / Contributions (incl. complex intervention design)	Level	Study Focus Area
Aarons GA et al., 2011	Advancing a conceptual model of evidence-based practice implementation in public service sectors	Framework Development	Exploration, Preparation, Implementation, Sustainment (EPIS) Framework	Proposes the EPIS (Exploration, Adoption/Preparation, Implementation, Sustainment) framework for implementing evidence-based practices in public service sectors; provides a staged, multi-level model that guides complex intervention design by mapping outer and inner context factors across the implementation lifecycle.	Meso	Implementation science
Adlan O et al., 2025	A scoping review of the use of behavioral theories in health professionals' continuing professional development research	Scoping Review	Behavioural Theories	Maps use of behavioural theories in CPD research; highlights gap between theory selection and rigorous application in designing professional development interventions; recommends explicit theoretical grounding at each intervention design stage.	Micro	Behaviour change application
Albrecht L et al., 2013	Development of a checklist to assess the quality of reporting of knowledge translation interventions using the WIDER recommendations	Guidance Development	WIDER Framework	Provides a checklist for reporting knowledge translation interventions based on WIDER recommendations; supports replication and fidelity assessment of complex interventions by standardising documentation of active ingredients, context, and delivery.	Meso	Implementation reporting
Ball H et al., 2025	How theories, models, and frameworks have been used to implement digital health interventions in services for people with severe mental health problems: a scoping review	Scoping Review	Multiple TMFs	Reviews how theories, models, and frameworks are applied in digital health implementation for severe mental health; identifies gaps in theory-driven design and highlights the need for explicit TMF integration across the digital intervention development process.	All levels	Digital health implementation
Barnden R et al., 2023	Prospective application of theoretical implementation frameworks to improve health care in hospitals - a systematic review	Systematic Review	Implementation Frameworks	Demonstrates that prospective (a priori) application of implementation frameworks improves intervention design quality, stakeholder engagement, and uptake in hospital settings; identifies barriers and facilitators to framework use during intervention planning.	Meso	Implementation science
Birken SA et al., 2017	Combined use of the Consolidated Framework for Implementation Research (CFIR) and the Theoretical Domains Framework (TDF): a systematic review	Systematic Review	CFIR + TDF Combined	Synthesises evidence on combining CFIR and TDF in implementation research; combined use strengthens multi-level analysis of barriers and facilitators and supports more comprehensive complex intervention design across inner and outer context levels.	Meso	Implementation science
Brennan C et al., 2021	A systematic review of the intervention characteristics, and behavior change theory and techniques used in mother-daughter interventions targeting physical activity	Systematic Review	BCT Analysis	Identifies BCTs and theoretical approaches used in mother-daughter physical activity interventions; highlights the importance of explicitly linking theory to intervention components and BCT selection during co-design of behaviour change interventions.	Micro	Behaviour change application
Cairns P et al., 2021	Interventions for the well-being of healthcare workers during a pandemic or other crisis: scoping review	Scoping Review	Wellbeing Frameworks	Identifies individual, team, and organisational interventions supporting healthcare worker wellbeing in crises; highlights need for multi-level, co-designed complex interventions that are context-sensitive and rapidly deployable during system disruption.	Meso	Workforce wellbeing
Cane J et al., 2012	Validation of the theoretical domains framework for use in behaviour change and implementation research	Validation Study	Theoretical Domains Framework	Validates the TDF as a reliable tool for identifying behavioural determinants; confirms its utility as a theoretical foundation for diagnosing barriers and informing BCT selection in complex intervention design for both behaviour change and implementation contexts.	Micro	Behaviour change
Colquhoun HL et al., 2017	Methods for designing interventions to change healthcare professionals' behaviour: a systematic review	Systematic Review	Multiple Behaviour Change Frameworks	Systematically reviews methods used to design behaviour change interventions for healthcare professionals; recommends integrating diagnostic analysis, theory selection, and iterative piloting as core stages in rigorous complex intervention design.	Micro	Intervention design methods
Cowdell F et al., 2019	How is the theoretical domains framework applied to developing health behaviour interventions? A systematic search and narrative synthesis	Systematic Review	Theoretical Domains Framework	Reviews how TDF is applied across the intervention development pipeline; finds TDF most commonly used for barrier/facilitator identification, with underuse in actual intervention component design and BCT selection; recommends fuller TDF integration across all design stages.	Micro	Behaviour change application
Duffy A et al., 2022	The Challenges Toward Real-world Implementation of Digital Health Design Approaches: Narrative Review	Narrative Review	Digital Health Design	Reviews real-world implementation challenges for digital health design approaches; identifies key barriers at organisational, technical, and user levels; highlights the need for iterative, context-sensitive design processes that account for implementation realities from the outset.	Meso/Macro	Digital health implementation
Dyson J et al., 2021	How is the Theoretical Domains Framework applied in designing interventions to support healthcare practitioner behaviour change? A systematic review	Systematic Review	Theoretical Domains Framework	Examines TDF application specifically for healthcare practitioner behaviour change interventions; identifies best-practice pathway from TDF-informed needs assessment to BCT selection and intervention logic model development.	Micro	Behaviour change application
Fischer M et al., 2021	Approach to Human-Centered, Evidence-Driven Adaptive Design (AHEAD) for Health Care Interventions: a Proposed Framework	Framework Development	AHEAD Framework	Proposes AHEAD framework integrating human-centred design with evidence-based practice for healthcare intervention development; provides structured iterative process for contextualised, user-centred complex intervention design including co-design, rapid prototyping, and real-world testing cycles.	All levels	Adaptive design
Fylan B et al., 2021	Using experience-based co-design with patients, carers and healthcare professionals to develop theory-based interventions for safer medicines use	Application Study	Co-design + Behaviour Change Theory	Demonstrates integration of experience-based co-design (EBCD) with behaviour change theory to develop complex interventions; shows how co-design surfaces implementation barriers and enables theory-informed selection of intervention strategies grounded in lived experience.	Micro/Meso	Participatory design
Gilhooly D et al., 2019	Barriers and facilitators to the successful development, implementation and evaluation of care bundles in acute care in hospital: a scoping review	Scoping Review	Implementation Frameworks	Identifies barriers and facilitators across the full care bundle lifecycle; underscores the importance of context assessment, stakeholder engagement, and piloting during complex intervention design to ensure implementability in acute care settings.	Meso	Implementation science
Godhwani K et al., 2025	Deliberative dialogue for co-design, co-implementation and co-evaluation of health-promoting interventions: a scoping review protocol	Scoping Review Protocol	Deliberative Dialogue	Presents protocol for reviewing deliberative dialogue as a method for co-designing, co-implementing, and co-evaluating complex health interventions; positions deliberative approaches as enhancing equity and stakeholder ownership throughout the intervention design lifecycle.	Meso	Participatory design
Gray P et al., 2019	Workplace-Based Organizational Interventions Promoting Mental Health and Happiness among Healthcare Workers: A Realist Review	Realist Review	Organisational Intervention Theory	Uses realist synthesis to identify mechanisms by which workplace organisational interventions improve healthcare worker mental health; highlights importance of designing interventions that are sensitive to organisational context, managerial support, and implementation conditions.	Meso	Workforce wellbeing
Green T et al., 2020	Use and reporting of experience-based codesign studies in the healthcare setting: a systematic review	Systematic Review	Co-design Methods	Reviews the use and quality of reporting of EBCD studies; identifies key design principles including patient narrative collection, co-design events, and joint priority-setting as integral steps in developing patient-centred complex interventions.	Meso	Participatory design

Author(s) & Year	Title	Type of Review	Primary Theoretical Approach	Key Findings / Contributions (incl. complex intervention design)	Level	Study Focus Area
Greenhalgh T et al., 2017	Beyond adoption: a new framework for theorizing and evaluating non-adoption, abandonment, and challenges to the scale-up, spread, and sustainability of health and care technologies	Framework Development	NASSS Framework	Introduces NASSS framework to explain technology non-adoption, abandonment, and scale-up failure; provides a multi-level analytical tool for anticipating and designing around implementation challenges in complex health technology interventions.	All levels	Technology implementation
Hailey V et al., 2022	A systematic review of behaviour change techniques used in interventions to increase physical activity among breast cancer survivors	Systematic Review	BCT Analysis	Systematically reviews BCTs used in physical activity interventions for breast cancer survivors; identifies most effective technique combinations to inform evidence-based selection of BCTs during complex intervention design for cancer survivorship populations.	Micro	Behaviour change application
Hamilton FL et al., 2025	Design and deployment of digital health interventions to reduce the risk of the digital divide and to inform development of the living with COVID recovery: a systematic scoping review	Systematic Scoping Review	Digital Health Design Frameworks	Reviews design and deployment approaches for digital health interventions addressing the digital divide; identifies equity-oriented design principles and co-production methods essential for developing inclusive complex digital interventions in recovery contexts.	Meso/Macro	Digital health implementation
Harvey G et al., 2016	PARIHS revisited: from heuristic to integrated framework for the successful implementation of knowledge into practice	Framework Development	PARIHS Framework	Revises PARIHS into an integrated framework (i-PARIHS) emphasising facilitation as the active element for implementing evidence into practice; provides structured approach for diagnosing context and tailoring implementation strategies within complex intervention design.	Meso	Implementation science
Hodkinson A et al., 2025	Effectiveness of different intervention designs for improving physical activity in adults with cardiometabolic conditions over time: a systematic review and network meta-analysis	Systematic Review / Meta-analysis	Multiple BCT Approaches	Network meta-analysis comparing intervention design features for physical activity in cardiometabolic populations; identifies most effective BCT combinations and delivery modalities; informs evidence-based selection of intervention components during complex intervention design.	Micro	Behaviour change application
Holt L et al., 2025	The person-based approach to intervention development: A scoping review of methods and applications	Scoping Review	Person-Based Approach	Reviews the Person-Based Approach (PBA) to intervention development; emphasises integration of qualitative evidence and user perspectives from earliest design stages to ensure complex interventions are acceptable, feasible, and optimised before evaluation.	Micro/Meso	Participatory design
Jackson B et al., 2024	FAIRSTEPS study - Framework Addressing Inequities in pRimary care using STakEholder PerspectiveS: Integrative evidence review and Delphi consensus	Integrative Review + Delphi	Health Equity Frameworks	Develops FAIRSTEPS framework addressing health inequities in primary care through stakeholder engagement; demonstrates how equity considerations and diverse stakeholder perspectives should be embedded within complex intervention design from the outset.	Meso/Macro	Health equity
Klaic M et al., 2022	Implementability of healthcare interventions: an overview of reviews and development of a conceptual framework	Overview of Reviews	Implementability Framework	Develops a conceptual framework for intervention implementability encompassing acceptability, feasibility, fidelity, and sustainability; provides practical guidance for assessing and building implementability into complex intervention design prior to trial.	Meso/Macro	Implementation science
Kok MC et al., 2015	Which intervention design factors influence performance of community health workers in low- and middle-income countries? A systematic review	Systematic Review	Community Health Worker Frameworks	Identifies intervention design factors (selection, training, supervision, incentives) that influence CHW performance in LMICs; provides evidence-based design principles for complex workforce interventions in low-resource health systems.	Meso	Workforce/health systems
May CR et al., 2018	Using Normalization Process Theory in feasibility studies and process evaluations of complex healthcare interventions: a systematic review	Systematic Review	Normalization Process Theory	Reviews NPT application in feasibility and process evaluation studies; demonstrates how NPT constructs (coherence, cognitive participation, collective action, reflexive monitoring) can structure formative evaluation to optimise complex intervention design before scale-up.	Meso	Implementation science
Michie S et al., 2013	The behaviour change technique taxonomy (v1) of 93 hierarchically clustered techniques: building an international consensus for the reporting of behaviour change interventions	Taxonomy Development	BCT Taxonomy	Establishes a gold-standard taxonomy of 93 BCTs organised into 16 clusters; provides a common language for specifying, reporting, and replicating the active components of complex behaviour change interventions, enabling cumulative science in intervention design.	Micro	Behaviour change
Michie S et al., 2011	The behaviour change wheel: a new method for characterising and designing behaviour change interventions	Framework Development	Behaviour Change Wheel	Introduces the Behaviour Change Wheel (BCW) and COM-B model as an integrated system for characterising behaviour and selecting appropriate intervention functions and policies; provides a structured, theory-linked method for designing behaviour change components of complex interventions.	Micro	Behaviour change
Movsisyan A et al., 2019	Adapting evidence-informed complex population health interventions for new contexts: a systematic review of guidance	Systematic Review	Adaptation Frameworks	Synthesises guidance for adapting complex interventions to new populations and contexts; identifies core adaptation steps including context analysis, stakeholder engagement, fidelity-adaptation balance, and re-piloting as essential for maintaining intervention effectiveness.	All levels	Intervention adaptation
Murray E et al., 2010	Normalisation process theory: a framework for developing, evaluating and implementing complex interventions	Framework Development	Normalisation Process Theory	Introduces NPT as a sociological framework for understanding how complex interventions become embedded in practice; provides theoretical grounding for designing interventions that account for organisational adoption, implementation work, and long-term normalisation.	All levels	Implementation science
Nguyen-Trung K et al., 2025	SeCOM-B: an integrated model for understanding human behaviour change in wicked socio-ecological problems	Framework Development	SeCOM-B Model	Extends COM-B to socio-ecological contexts through the SeCOM-B model; integrates systems thinking and behaviour change theory to inform design of complex interventions addressing wicked problems where individual, social, and environmental determinants interact.	All levels	Systems behaviour change
Noyes J et al., 2014	Reconceptualizing children's complex discharge with health systems theory: novel integrative review with embedded expert consultation and theory development	Integrative Review	Health Systems Theory	Reconceptualises paediatric complex discharge using health systems theory; demonstrates how systems-level theorising can reframe intervention design problems, revealing multi-level leverage points and interdependencies not visible at the individual care level.	Macro	Health systems
O'Cathain A et al., 2019	Guidance on how to develop complex interventions to improve health and healthcare	Guidance Development	Complex Intervention Frameworks	Provides comprehensive, updated guidance on the full complex intervention development process including problem analysis, intervention theory, evidence synthesis, co-design, piloting, and evaluation planning; represents current consensus on best-practice intervention design.	All levels	Intervention design
Prasad SK et al., 2025	Interventions to improve hand hygiene in community settings: a systematic review of theories, barriers and enablers, behaviour change techniques and hand hygiene station design features	Systematic Review	Behaviour Change Theory + WASH Frameworks	Reviews theories, barriers/enablers, BCTs, and physical design features used in community hand hygiene interventions; provides an integrated evidence base linking theoretical frameworks to concrete intervention design decisions including environmental and infrastructure components.	Micro/Meso	Behaviour change application
Raudasoja AJ et al., 2022	Randomized controlled trials in de-implementation research: a systematic scoping review	Systematic Scoping Review	De-implementation Frameworks	Reviews RCT designs in de-implementation research; identifies design considerations specific to reducing or stopping low-value practices, including theory selection, context assessment, and outcome measurement, which mirror but differ from standard complex intervention design.	All levels	De-implementation
Reid H et al., 2022	Use of the behaviour change wheel to improve everyday person-centred conversations on physical activity across healthcare	Application Study	Behaviour Change Wheel	Demonstrates BCW application to design and deliver a person-centred physical activity intervention in clinical consultations; illustrates how BCW and COM-B can structure needs assessment, BCT selection, and healthcare professional training as integrated intervention components.	Micro	Behaviour change application
Skivington K et al., 2021	From complex social interventions to interventions in complex social systems: future directions and unresolved questions for intervention development and evaluation	Guidance Development	MRC Framework	Updates the MRC framework for complex interventions, reframing the focus from complicated interventions to interventions within complex adaptive systems; provides guidance on using systems thinking, stakeholder co-production, and realist evaluation in intervention development and evaluation.	All levels	Intervention design

Author(s) & Year	Title	Type of Review	Primary Theoretical Approach	Key Findings / Contributions (incl. complex intervention design)	Level	Study Focus Area
Soames J et al., 2024	Electronic health record-based behaviour change interventions aimed at general practitioners in the UK: a mixed methods systematic review using behaviour change theory	Mixed Methods Review	Behaviour Change Theory	Reviews EHR-based behaviour change interventions targeting GPs; applies behaviour change theory to understand design features influencing uptake; highlights importance of system-level design factors alongside individual BCTs in digital behaviour change intervention design.	Micro/Meso	Behaviour change application
Vasan A et al., n.d.	Support and performance improvement for primary health care workers in low- and middle-income countries: a scoping review of intervention design and methods	Scoping Review	Health Worker Performance Frameworks	Reviews intervention design and methods for supporting primary health care workers in LMICs; identifies key design features including training modalities, supervision structures, and incentive mechanisms essential to effective complex workforce intervention design in resource-limited settings.	Meso	Workforce/health systems
Wallner M et al., 2023	Theory-based evaluation and programme theories in nursing: discussion on the updated Medical Research Council framework	Discussion/Framework Review	MRC Framework	Discusses updated MRC framework in relation to theory-based evaluation and programme theory development in nursing; emphasises the role of explicit logic models and programme theories in articulating how and why complex interventions are expected to produce change.	All levels	Implementation science
Webb J et al., 2022	The barriers and facilitators to physical activity in people with a musculoskeletal condition: A rapid review of reviews using the COM-B model to support intervention development	Rapid Review	COM-B Model	Uses COM-B to synthesise barriers and facilitators to physical activity in MSK populations; demonstrates rapid review methodology as a theory-driven approach to needs assessment that directly informs BCT selection and intervention component design.	Micro	Behaviour change application
Whelan J et al., 2023	Combining systems thinking approaches and implementation science constructs within community-based prevention: a systematic review	Systematic Review	Systems Thinking	Identifies how systems thinking approaches (e.g., CAS, systems dynamics) can be integrated with implementation science constructs to design community-based prevention interventions; highlights value of understanding feedback loops and emergent properties during intervention design.	Macro	Systems thinking
Willis CD et al., 2016	Scaling up complex interventions: insights from a realist synthesis	Realist Synthesis	Systems Thinking	Uses realist synthesis to identify mechanisms underpinning successful scale-up of complex interventions; reveals how contextual factors, stakeholder relationships, and adaptive implementation strategies must be built into intervention design to support spread and sustainability.	Macro	Scale and spread
