# Supplementary material for: Designing complex health system interventions: an integrated theory-informed approach
Source: Front Health Serv. 2026 Jul 9;6:1820296. doi: 10.3389/frhs.2026.1820296 (PMC13391582; doi:10.3389/frhs.2026.1820296)
Supplement: Supplementary file 2 [file Table1.pdf]

Table 1. Theories, models, and frameworks (TMFs) in mini review: tradition, level of action within health system, contribution to intervention design, examples of operationalisation, and conceptualisation of complexity

Theory, model, or framework (TMF)	Tradition	Primary level	Use during design	Key contribution to design	Example of how the TMF has been operationalised (drawn from the included reviews)	How TMF conceptualises complexity
**COM-B / Behaviour Change Wheel (BCW)** *Michie et al., 2011 [17]*	**Behavioural**	Micro	Prospective	Diagnoses behavioural determinants (Capability, Opportunity, Motivation) and links them to candidate intervention functions and BCTs.	Webb et al. (2022) [24] used COM-B to synthesise barriers and facilitators to physical activity in musculoskeletal populations and align determinants with intervention techniques. Reid et al. (2022) [27] used the BCW to structure person-centred physical activity conversations in clinical consultations.	Locates complexity in the individual; intervention functions account for environmental opportunity, but feedback, emergence and cross-level dynamics are not modelled.
**Theoretical Domains Framework (TDF)** *Cane et al., 2012 [18]*	**Behavioural**	Micro–Meso	Prospective and retrospective	Maps behavioural determinants across 14 domains; widely used to structure healthcare professional behaviour change.	Dyson & Cowdell (2021) [31] and Cowdell & Dyson (2019) [32] reviewed TDF applications across the design pipeline; TDF was most often used for barrier/facilitator identification, with under-use in actual component design and BCT selection.	Acknowledges environmental, social and resource influences as separate domains; interactions between domains are not formally theorised.
**Behaviour Change Technique (BCT) Taxonomy** *Michie et al., 2013 [23]*	**Behavioural**	Micro	Prospective and retrospective	Provides a shared taxonomy of 93 BCTs to specify intervention content reproducibly.	Brennan et al. (2021) [26] coded BCTs in mother-daughter physical activity interventions; Hailey et al. (2022) [25] coded BCTs in breast cancer survivor physical activity interventions; Hodkinson et al. (2025) [28] linked BCT combinations to outcomes in a network meta-analysis.	Atheoretical descriptor system; complexity is not engaged at the taxonomy level but downstream during use.
**Health Belief Model; Theory of Planned Behaviour; Social Cognitive Theory** *Rosenstock 1974 [21]; Ajzen 1991 [19]; Bandura 1986 [20]*	**Behavioural**	Micro	Prospective	Foundational theories of motivation, self-efficacy and reasoned action that underpin many modern behavioural frameworks.	Underpin the constructs in COM-B, TDF and the BCT Taxonomy; used implicitly within the included reviews rather than directly as standalone design tools.	Linear, individual-level causal models; complexity is largely absent.
**Transtheoretical Model / Stages of Change** *Prochaska & DiClemente, 1986 [22]*	**Behavioural**	Micro	Prospective	Frames behaviour change as a stage-based process (pre-contemplation → contemplation → preparation → action → maintenance); supports tailoring of intervention components to readiness-to-change.	Embedded within wider behaviour-change designs reviewed in the included literature where readiness-to-change is a tailoring parameter; commonly used implicitly within COM-B and BCT-based interventions rather than as a standalone design tool.	Captures temporal dynamics of individual behaviour change; multi-actor and system feedback are not addressed.
**Behavioural theory in continuing professional development and EHR-supported care** *Bartholomew & Mullen, 2011 [12]; Prasad et al., 2025 [30]; Soames & Pettigrew, 2024 [29]; Adlan et al., 2025 [33]; & Colquhoun et al., 2017 [34].*	**Behavioural**	Micro–Meso	Prospective and retrospective	Applies behavioural theories and multi-framework approaches to professional behaviour change, intervention design methods, and population behaviour interventions.	Adlan et al. (2025) [33] mapped theory uses in CPD; Soames & Pettigrew (2024) [29] reviewed EHR-based behaviour change for general practitioners; Colquhoun et al. (2017) [34] synthesised methods for designing professional behaviour change; Prasad et al. (2025) [30] applied multiple theoretical approaches to community hand hygiene; Bartholomew & Mullen (2011) [12] articulated five roles of theory in design and testing.	Surfaces multi-level determinants but treats them as discrete; relational and emergent system properties not formally modelled.
**Combined CFIR + TDF** *Birken et al., 2017 [51]*	**Hybrid (behaviour + implementation)**	Micro–Meso	Prospective	Integrates contextual (CFIR) and behavioural (TDF) determinants in a single analytic strategy; bridges organisational context and individual behaviour within one analysis.	Birken et al. (2017) [51] synthesised studies that paired CFIR with TDF to capture multi-level determinants of implementation; combined use enables design teams to address contextual and behavioural drivers concurrently.	Hybrid framework that explicitly bridges two traditions - an exemplar of the overlap-zone TMFs that respond to calls for cross-tradition integration.
**Person-Based Approach (PBA)** *Holt et al., 2025 [35]*	**Hybrid (behaviour + implementation)**	Micro–Meso	Prospective	Integrates qualitative user research with theory-based design; common in digital intervention development.	Holt et al. (2025) [35] reviewed PBA applications and showed how qualitative user perspectives, integrated from the earliest design stages, ensure interventions are acceptable, feasible and optimised before evaluation.	Engages contextual variability through user perspectives; system-level dynamics remain implicit.

Theory, model, or framework (TMF)	Tradition	Primary level	Use during design	Key contribution to design	Example of how the TMF has been operationalised (drawn from the included reviews)	How TMF conceptualises complexity
**Co-design / experience-based co-design with behaviour change theory** *Green et al., 2020 [36]; Fylan et al., 2021 [37]*	**Hybrid (behaviour + implementation)**	Meso	Prospective and iterative	Combines participatory co-design methods with behaviour change theory and quality improvement to develop context-sensitive interventions.	Green et al. (2020) [36] reviewed experience-based co-design across healthcare settings; Fylan et al. (2021) [37] applied theory-based co-design to medicines safety, integrating co-design with behaviour change theory in intervention development.	Engages relational and contextual complexity through participatory practice; does not formally model system dynamics.
**Normalization Process Theory (NPT)** *May et al., 2018 [42]; May, 2013 [6]*	**Hybrid (behaviour + implementation)**	Meso	Prospective	Four constructs (coherence, cognitive participation, collective action, reflexive monitoring) explain how interventions become embedded in routine practice.	May et al. (2018) [42] reviewed NPT use in feasibility studies; teams used the four constructs to identify and reduce cognitive burden, clarify role expectations across clinicians and facilitators, and restructure intervention components to align with routine clinical pathways.	Bridges sociology, behaviour and organisation; theorises relational and processual complexity but stops short of formal feedback modelling.
**Consolidated Framework for Implementation Research (CFIR)** *Damschroder et al., 2009 [43]*	**Implementation science**	Meso–Macro	Mostly retrospective; increasingly prospective	Structured taxonomy of inner setting, outer setting, intervention, and individual determinants; supports context-sensitive design.	Used prospectively to inform adaptations such as flexible delivery formats and embedded implementation supports; Barnden et al. (2023) [52] demonstrated prospective CFIR use in hospitals enhanced design quality, stakeholder engagement and uptake.	Treats determinants as discrete variables; emergent and feedback properties not formally modelled.
**EPIS (Exploration, Preparation, Implementation, Sustainment)** *Aarons et al., 2011 [44]*	**Implementation science**	Meso–Macro	Prospective	Phases the implementation lifecycle; structures temporal sequencing of intervention design and rollout.	Used as a planning scaffold across the implementation lifecycle; commonly applied to structure design and rollout sequencing in evidence-based practice implementation.	Captures temporal complexity across phases; cross-level feedback is not explicit.
**RE-AIM Framework** *Glasgow et al., 1999 [45]*	**Implementation science**	Meso–Macro	Mostly retrospective; increasingly prospective	Evaluates public health impact across Reach, Effectiveness, Adoption, Implementation, Maintenance; surfaces heterogeneity of impact across actors and settings.	Used widely to structure evaluation; among the most commonly used implementation frameworks in systems-thinking community-based prevention studies.	Surfaces heterogeneity of impact across actors and settings; does not formally model dynamic interactions or feedback.
**i-PARIHS (integrated Promoting Action on Research Implementation in Health Services)** *Harvey & Kitson, 2016 [46]*	**Implementation science**	Meso	Prospective	Foregrounds facilitation as the active ingredient mediating between innovation, recipients, and context.	Applied in implementation projects to plan the facilitation role and target the interaction between innovation, recipients, and context, particularly in nursing and primary-care settings.	Recognises contextual interaction and relational complexity; less formal treatment of system dynamics.
**Implementation theories, frameworks, and selection guidance** *Nilsen, 2015 [9]; Lynch et al., 2018 [15]; Moore & Evans, 2017 [8]; Davidoff et al., 2015 [10]; De Silva et al., 2014 [11]*	**Implementation science**	Meso–Macro	Prospective	Categorise implementation theories, guide pragmatic theory selection, demystify theory use in improvement, and integrate theory of change with the MRC framework.	Nilsen (2015) [9] categorises implementation theories; Lynch et al. (2018) [15] provide a pragmatic guide for selecting theoretical approaches; Moore & Evans (2017) [8] address theory selection for population health; Davidoff et al. (2015) [10] demystify theory use in improvement science; De Silva et al. (2014) [11] enhance the MRC framework with theory of change.	Acknowledges multi-level determinants; theory-of-change variants make programme logic explicit but rarely model system feedback.
**Implementability Framework** *Klaic et al., 2022 [53]*	**Implementation science**	Meso–Macro	Prospective	Conceptual framework that structures judgements about whether an intervention can feasibly be implemented in practice.	Klaic et al. (2022) [53] synthesised reviews to derive an implementability framework, integrating systems-thinking considerations into prospective intervention design.	Acknowledges systems-level constraints on implementation; treats them as structured determinants rather than dynamic interactions.
**Implementation frameworks applied prospectively in hospital settings** *Barnden et al., 2023 [52]*	**Implementation science**	Meso	Prospective	Demonstrates prospective application of implementation frameworks to hospital-based intervention design and quality improvement.	Barnden et al. (2023) [52] reviewed prospective use of implementation frameworks in hospitals, showing enhanced stakeholder engagement, design quality and uptake when frameworks are embedded from the outset.	Captures organisational determinants; system dynamics remain implicit.
**Theory use in digital mental health interventions** *Ball et al., 2025 [41]*	**Implementation science**	All levels	Prospective and retrospective	Maps the use of theories, models and frameworks across digital mental health intervention design and implementation.	Ball et al. (2025) [41] conducted a scoping review of TMF use in digital mental health, surfacing inconsistent theory application across the development–implementation pipeline.	Surfaces multi-level theory use; engages digital-context complexity but does not formally model it.

Theory, model, or framework (TMF)	Tradition	Primary level	Use during design	Key contribution to design	Example of how the TMF has been operationalised (drawn from the included reviews)	How TMF conceptualises complexity
**NASSS framework (Non-adoption, Abandonment, Scale-up, Spread, Sustainability)** *Greenhalgh et al., 2017 [13]*	**Hybrid (implementation + complexity)**	Cross-cutting	Prospective	Anticipates where complexity will threaten adoption and scale across seven domains for technology-enabled complex interventions.	Used to anticipate complexity-related adoption challenges across condition, technology, value proposition, adopters, organisation, wider system, and embedding/adaptation over time.	Explicitly built on complexity science; operationalises non-linearity, emergent challenges, and adaptation across domains.
**Complexity science principles for healthcare** *Plsek & Greenhalgh, 2001 [2]; Greenhalgh & Papoutsi, 2018 [3]*	**Systems / complexity**	All levels	Prospective (paradigm-setting)	Introduces complexity science to healthcare and calls for a paradigm shift toward complexity-informed health services research.	Plsek & Greenhalgh (2001) [2] introduced complexity principles to a clinical audience; Greenhalgh & Papoutsi (2018) [3] argued for complexity-informed health services research as a paradigm shift.	Frames complexity as the central unit of analysis; sets the conceptual ground for downstream operationalisation.
**Systems thinking for complex interventions and scale-up** *Moore et al., 2019 [7]; Willis et al., 2016 [16]*	**Systems / complexity**	All levels (Macro for scale-up)	Prospective	Distinguishes complex interventions from complex systems and provides realist insights into scaling up complex interventions.	Moore et al. (2019) [7] reframe evaluation by distinguishing the intervention from the system in which it acts; Willis et al. (2016) [16] use realist synthesis to surface mechanisms underlying successful scale-up.	Treats outcomes as emergent properties of intervention-system interaction; foundational to systems-informed evaluation.
**SEIPS 2.0 (Systems Engineering Initiative for Patient Safety)** *Holden et al., 2013 [63]*	**Systems / complexity**	Meso	Prospective	Models healthcare work as a sociotechnical system of people, tasks, tools, technology, environment, organisation, and outcomes; designed to anticipate how interventions interact with existing work systems.	Used during design to map healthcare work as a sociotechnical system and identify how interventions interact with existing tasks, tools, environment, and organisation; supports anticipation of unintended workflow effects.	Operationalises systems thinking through structured sociotechnical mapping; surfaces interdependencies between work-system elements.
**Engineering Better Care (EBC)** *Royal Academy of Engineering, 2017 [64]*	**Systems / complexity**	Meso–Macro	Prospective	Adapts systems engineering principles for health and care system design; relies on stakeholder-engaged systems mapping (flowcharts, causal loop diagrams) to inform design choices.	Used through stakeholder-engaged systems mapping to convert complex information about a health system into a shared visual representation that informs prospective design decisions.	Directly operationalises system structure, boundaries, interactions, and points of intervention.
**System Dynamics / Causal Loop Diagrams and Agent-Based Modelling** *Lich et al., 2017 [58]; Tracy et al., 2018 [59]*	**Systems / complexity**	Macro	Prospective / formative	Formal computational and diagrammatic modelling of stocks, flows, feedback loops and agent interactions; supports scenario testing, leverage-point identification and policy analysis.	Applied to model how interventions interact with system structure over time and to simulate how heterogeneous actors' behaviour produces population-level outcomes; supports exploration of intervention scenarios before real-world trial.	Most explicit operationalisation of feedback, accumulation, delay, non-linearity, emergence, and heterogeneity in formal computational form.
**Group model building / participatory systems modelling** *Canty et al., 2025 [72]*	**Systems / complexity**	Macro	Prospective	Builds stakeholder capacity for systems thinking through collaborative modelling; positions systems modelling as a participatory practice that enhances implementation of public health interventions.	Canty et al. (2025) [72] demonstrated the use of system dynamics modelling with stakeholders to build capacity for systems thinking and enhance the implementation of public health interventions in the opioid response context.	Operationalises systems thinking as a participatory practice rather than a purely technical method; surfaces feedback and interdependencies through stakeholder dialogue.
**Realist methods (Context–Mechanism–Outcome configurations)** *Fletcher et al., 2016 [60]*	**Systems / complexity**	Cross-cutting	Prospective and retrospective	Context–Mechanism–Outcome (CMO) configurations explain for whom, how and under what circumstances interventions work; aligns with complexity by treating outcomes as emergent from context–mechanism interaction.	Used in realist syntheses to identify mechanisms by which interventions produce outcomes under different contextual conditions; surfaces how outcomes depend on contextual moderators and implementation conditions.	Treats outcomes as emergent from context-mechanism interaction; aligns with complexity but does not formally model system dynamics.
**SeCOM-B (Socio-ecological COM-B)** *Nguyen-Trung et al., 2025 [40]*	**Hybrid (behaviour + systems / design)**	Cross-cutting	Prospective	Extends COM-B into socio-ecological systems for wicked problems where individual, social and environmental determinants interact.	Nguyen-Trung et al. (2025) [40] propose SeCOM-B as an integrated model for understanding human behaviour change in wicked socio-ecological problems.	Hybrid framework that explicitly imports systems thinking into a behaviour change model.
**Design thinking + systems science + implementation science integration** *Huang et al., 2024[77]*	**Hybrid (behaviour + systems / design)**	Cross-cutting	Prospective	Argues for solution-oriented integration of design thinking, systems science, and implementation science to advance	Huang et al. (2024) [77] propose a framework that integrates design thinking, systems science, and implementation science, providing an explicit three-way integration directly responsive to calls for	Explicit three-way integration; positions design practice in the overlap zone between behavioural,

Theory, model, or framework (TMF)	Tradition	Primary level	Use during design	Key contribution to design	Example of how the TMF has been operationalised (drawn from the included reviews)	How TMF conceptualises complexity
				implementation science from a problem-oriented to a solution-oriented paradigm.	cross-tradition synthesis (referenced in the wider literature on integrative TMFs).	implementation and systems traditions.
**Approach to Human-Centered, Evidence-Driven Adaptive Design (AHEAD)**<br>*Fischer et al., 2021 [38]*	**Hybrid (behaviour + systems / design)**	Cross-cutting	Prospective and iterative	Iterative prototyping responsive to context; integrates evidence with human-centred design.	Fischer et al. (2021) [38] developed AHEAD as a structured iterative process for contextualised, user-centred complex intervention design including co-design, rapid prototyping, and real-world testing cycles.	Operationalises adaptation and iteration as a response to system dynamics.
**Health Systems Framework**<br>*Jamison et al., 2006 [1]*	**Cross-cutting guidance / reporting**	Macro	Reference framework	Defines foundational health system pillars and components, providing the macro-level structure for intervention design.	Jamison et al. (2006) [1] articulate the health system pillars used as the macro-level scaffolding for many subsequent intervention design efforts.	Structures the system into components; does not model dynamic interactions.
**MRC framework for complex interventions**<br>*Skivington et al., 2021 [4]*	**Cross-cutting guidance / reporting**	Cross-cutting	Prospective	Guides the full development–evaluation lifecycle; the 2021 update emphasises programme theory, stakeholders, and context.	Used as a high-level scaffold for intervention development and evaluation; the 2021 update emphasises iterative use of programme theory, stakeholder engagement and contextual adaptation.	Explicitly accommodates complexity perspectives in the 2021 update; organises rather than models dynamic properties.
**MRC ADAPT and adaptation guidance**<br>*Moore et al [5], Movsisyan et al., 2019 [14]*	**Cross-cutting guidance / reporting**	Cross-cutting	Prospective	Structured guidance for adapting evidence-informed interventions to new contexts.	Movsisyan et al. (2019) [14] reviewed adaptation frameworks and produced guidance for adapting interventions across populations and settings.	Directly addresses contextual variability and adaptation as core features of complexity.
**Workgroup for Intervention Development and Evaluation Research (WIDER) recommendations**<br>*Albrecht et al., 2013 [39]*	**Cross-cutting guidance / reporting**	Cross-cutting	Reporting standard	Standardises documentation of intervention components and context to support replication.	Albrecht et al. (2013) [39] developed a checklist that supports standardised reporting of active ingredients, context, and delivery, enabling fidelity assessment and replication of complex interventions.	Improves transparency in reporting complexity but does not theorise it.
